# Distribution of *Dehalococcoidia* in the Anaerobic Deep Water of a Remote Meromictic Crater Lake and Detection of *Dehalococcoidia*-Derived Reductive Dehalogenase Homologous Genes

**DOI:** 10.1371/journal.pone.0145558

**Published:** 2016-01-06

**Authors:** Corinne Biderre-Petit, Eric Dugat-Bony, Mickaël Mege, Nicolas Parisot, Lorenz Adrian, Anne Moné, Jérémie Denonfoux, Eric Peyretaillade, Didier Debroas, Delphine Boucher, Pierre Peyret

**Affiliations:** 1 Laboratoire “Microorganismes: Génome et Environnement,” Clermont Université, Université Blaise Pascal, F-63000, Clermont-Ferrand, France; 2 Laboratoire Microorganismes, Génome et Environnement, Centre National de la Recherche Scientifique (CNRS), Unité Mixte de Recherche (UMR) 6023, F-63171, Aubière, France; 3 UMR GMPA, AgroParisTech, INRA, Université Paris-Saclay, 78850, Thiverval-Grignon, France; 4 Clermont Université, Université d’Auvergne, EA 4678 CIDAM, BP 10448, F-63001, Clermont-Ferrand, France; 5 Helmholtz Centre for Environmental Research–UFZ, Permoserstraße 15, D-04318, Leipzig, Germany; Belgian Nuclear Research Centre SCK•CEN, BELGIUM

## Abstract

Here we describe the natural occurrence of bacteria of the class *Dehalococcoidia* (DEH) and their diversity at different depths in anoxic waters of a remote meromictic lake (Lake Pavin) using 16S rRNA gene amplicon sequencing and quantitative PCR. Detected DEH are phylogenetically diverse and the majority of 16S rRNA sequences have less than 91% similarity to previously isolated DEH 16S rRNA sequences. To predict the metabolic potential of detected DEH subgroups and to assess if they encode genes to transform halogenated compounds, we enriched DEH-affiliated genomic DNA by using a specific-gene capture method and probes against DEH-derived 16S rRNA genes, reductive dehalogenase genes and known insertion sequences. Two reductive dehalogenase homologous sequences were identified from DEH-enriched genomic DNA, and marker genes in the direct vicinity confirm that gene fragments were derived from DEH. The low sequence similarity with known reductive dehalogenase genes suggests yet-unknown catabolic potential in the anoxic zone of Lake Pavin.

## Introduction

Some of the microorganisms living in anoxic environments have the ability to reductively dehalogenate organochlorides. Indeed, anaerobic bacteria belonging to the *Firmicutes* (*Desulfitobacterium* and *Dehalobacter*), *Proteobacteria* (e.g. *Desulfomonile*, *Desulfuromonas* and *Sulfurospirillum*) and *Chloroflexi* (*Dehalococcoides*, *Dehalogenimonas* and ‘*Dehalobium*’, all affiliated to the class *Dehalococcoidia* (DEH)) [1] couple reductive dechlorination of organochlorides to energy conservation in a respiratory manner currently referred to as organohalide respiration (OHR). This process has been extensively studied because of its useful application in the bioremediation of soil and groundwater contaminated with chlorinated compounds such as tetrachloroethene (PCE) and trichloroethene (TCE) [2–4]. All DEH cultured thus far are obligate organohalide respirers and share similar limited metabolic capabilities using hydrogen as an electron donor and organohalogen compounds as electron acceptors [1,5,6]. The key enzymes for OHR are the reductive dehalogenases (RDases) (for a review see [7]). Of all types of dehalogenases discovered over the past few decades, RDases are the most studied due to their often observed capacity to transform highly halogenated compounds. RDases also attract attention because many of them are part of membrane-bound electron transport chains in organohalide-respiring anaerobic bacteria (OHRB) [8]. However, despite the increasing interest on transformation reactions of organochlorides, only a limited number of studies have focused on the degradation of compounds occurring naturally, and little is known about the extent and the ecological role of dechlorination in non-polluted environments [9–11].

Beyond their importance in the remediation of contaminated environments [3,12], the activities of DEH are also of fundamental interest in uncontaminated environments, where up to 2,300 organochlorides have been identified from biotic and abiotic natural sources [13]. Recently, with the advances of molecular methods (such as metagenomics and single-cell genomics), members of the DEH and many reductive dehalogenase homologous genes (*rdhA)* have been detected in various uncontaminated environments, ranging from soils [11,14] to sedimentary habitats from marine [10,15–18] and freshwater systems [14,19,20]. The widespread detection of DEH-affiliated 16S rRNA and *rdhA* gene sequences in these environments suggests the presence of OHR as an ecologically significant microbial activity. At the same time, the absence of canonical *rdhA* genes similar to those found in cultivated DEH strains as well as the evidence for additional metabolic lifestyles predicted from some DEH assembled genomes from these pristine environments [16,17,19] have shed light on the metabolic versatility of some DEH-related members. This suggests that reductive dehalogenation could be a recently acquired trait, not conserved within all DEH. Therefore, DEH populations might play an important role in the chlorine cycle as well as biogeochemical cycles, including the carbon, within uncontaminated environments [16].

While it is widely believed that microbial dehalogenation plays a significant role in the functioning of the halogen cycle, almost nothing is known about the ecological role and the metabolic characteristics of DEH in lake waters. The only data currently available come from studies aiming to describe the microbial diversity in the stratified waters of two meromictic lakes, Lake Sakinaw (Canada) [21] and Lake Kivu (East Africa) [22]. From these investigations, it has emerged that bacteria affiliated to DEH were widely distributed in the anoxic zone of these uncontaminated ecosystems. In meromictic lakes, the water column stably partitions into an oxic surface water zone (mixolimnion), a redox transition zone (mesolimnion) and an anoxic, often hydrogen sulfide and methane-rich bottom water zone (monimolimnion) [22]. These results suggest that presence of DEH may be an indicator for the monimolimnion.

Lake Pavin in the French Massif Central, the subject of our study, is a meromictic lake with permanent redox stratification and no direct industrial inflow [23]. We investigated the presence of DEH in the anoxic water column of Lake Pavin by using a combination of classical molecular approaches -including PCR, qPCR, metabarcoding, and shotgun sequencing- as well as recently established new molecular approaches -including specific-gene capture to isolate large genomic fragments. The aim of our study was to analyze the occurrence, distribution and diversity of DEH in the monimolimnion and their *rdhA* genes.

## Materials and Methods

### Study site: Lake Pavin

Lake Pavin is a small (~0.44 km^2^), deep (92 m), almost circular (~750 m diameter) lake located in the Massif Central (France) (45°29.740N, 2°53.280E), 35 km southwest of Clermont-Ferrand, at an altitude of 1,197 m above sea level. It occupies a maar crater which formed 6,900 years ago. Its drainage basin is densely covered by mixed deciduous/coniferous forest. This lake is well preserved from human activities because swimming, diving and motorboats are prohibited and no important road or industrialized town is in the immediate vicinity.

Lake Pavin is meromictic and therefore characterized by two stable stratified layers: i) an upper layer (mixolimnion) that spans from the surface to around 55 m depth which is oxygenated due to vertical seasonal mixing and ii) a deeper layer (monimolimnion) that spans from around 65 to 92 m depth which is permanently anoxic and never mixes with upper oxygenated waters. In the mixolimnion, the chemical profiles are shaped by the activity of photosynthetic organisms and associated heterotrophs. Oxygen (O_2_) is produced by photosynthesis and accumulates in the photic zone. In this area, nutrients such as nitrate (NO_3_^-^) and phosphate (PO_4_^3-^) are depleted due to biological assimilation while dissolved Fe and sulfate (SO_4_^2-^) concentrations are a few tens of nM and 15–20 μM, respectively [23–25].

In contrast, the deep anoxic monimolimnion is highly reducing and contains higher concentrations of almost all compounds except SO_4_^2-^, NO_3_^-^ and O_2_ than the mixolimnion. It contains high concentrations of dissolved ionic species, as shown by a strong increase in specific conductivity with depth [23]. The main dissolved ions in this area are bicarbonate (HCO_3_^-^), ferrous iron (Fe^2+^) and ammonium (NH_4_^+^) whereas the main uncharged species are carbon dioxide (CO_2_) and methane (CH_4_) [23,24,26,27]. Dissolved inorganic phosphorus and dissolved organic carbon are also highly concentrated and reaches up to 340 μM and 400 μM [26,28]. Sulfate is reduced to sulfide just below the oxycline with SO_4_^2-^ concentrations in the low μM levels (< 4 μM) (for review, see [23]). In spite of sublacustrine water inputs, the enrichment of most elements in bottom waters seems mostly to be due to internal cycling. On a decadal time scale, the available data have shown that the deep part of the lake has reached a steady state [29,30]. Mixolimnion and monimolimnion are separated by a third layer called mesolimnion which is characterized by sharp gradients of dissolved Fe and salinity.

### Sample collection

Water samples were obtained from a platform positioned above the deepest zone of the water column as previously described [31]. They were collected in March 2012 using an 8-L horizontal Van Dorn bottle along a vertical profile at depths with contrasting O_2_ and chemical conditions (52, 58, 60, 63, 68 and 80 m), after dissolved O_2_ measurement using a submersible multiparameter probe (YSI GRANT 3800, Cambridge, UK). The water column of Lake Pavin did not involve endangered or protected species thus no specific permissions were required for sample collection. Immediately upon collection, the water samples were transferred into sterile bottles and stored on ice (2–3 hrs maximum). The water samples were filtered in the laboratory (0.3–0.6 L of water per filter, depending on clogging rates) onto 47-mm TSTP Millipore filters (0.22 μm pore size) (Millipore, Billerica, Massachusetts, USA). Filters were stored at -80°C until nucleic acid extraction.

### Nucleic acid isolation, cDNA synthesis and pyrosequencing

Genomic DNA (gDNA) and total RNA were extracted from water samples as previously described [32], except at 52 m for total RNA, where all extraction attempts failed. The RNA was checked for integrity on a Model 2100 Bioanalyzer (Agilent Technologies, Santa Clara, California, USA) using the RNA 6000 Nano kit (Agilent Technologies, Santa Clara, CA, USA), whereas the DNA integrity was analyzed on an agarose gel. The 16S rRNA gene reverse transcription was performed from ~30 ng of total RNA using SuperScript III reverse transcriptase as suggested by the manufacturer (Invitrogen Inc., Carlsbad, CA, USA) with the primer 1492R (S1 Table) [33].

The amplification of the V1-V3 hypervariable regions of the 16S rRNA genes was performed by an external service (MR DNA, http://mrdnalab.com) using the 27F/Gray519R primer set (S1 Table) on all gDNA and cDNA samples, followed by pyrosequencing using a Roche 454 GS-FLX Titanium platform. The sequences were checked for the following quality criteria: no ambiguous nucleotides, a minimum quality score of 27 according to Pangea trimming [34], a minimum length of 200 bp and no sequencing error in the forward primer. Chimeras were detected using the UCHIME program [35] and removed before further analysis (1.3% of reads). The remaining reads were clustered in operational taxonomic units (OTUs) at a 97% similarity threshold. The process was automated by PANAM, which also computed the richness and diversity indexes (https://code.google.com/p/panam-phylogenetic-annotation/) [36].

#### Clone libraries and phylogenetic analyses

The 16S rRNA amplicons obtained with specific PCR primer sets for different OHRBs (S1 Table) on the gDNA and cDNA samples were cloned using the TOPO TA cloning kit (Invitrogen Inc., Carlsbad, CA, USA) and sequenced using the Sanger sequencing technology by Eurofins Genomics sequencing services (https://eurofinsgenomics.eu). Sequence similarities were identified using the online NCBI BLASTn [37]. Phylogenies were constructed with MEGA6 software [38] using the maximum composite-likelihood model and neighbor-joining algorithm. All 16S rRNA sequences were submitted to GenBank (accession numbers KJ475586 to KJ475644 and KJ475666 to KJ475673).

### Real-time PCR

Real-time PCR assays (qPCR) were performed on the gDNA samples using a Mastercycler Epgradients realplex (Eppendorf, Hamburg, Germany) and data analysis was done with Realplex software (version 1.5). The primer sets used in this study are listed in S1 Table. Briefly, the PCR reactions (each conducted in triplicate) contained 12.5 μL of 2X MESA GREEN qPCR MasterMix Plus for SYBR Assay® (Eurogentec S.A, Liege, Belgium), 1 μL of DNA template (pure or diluted 1:100), 1.75 μl of each primer (0.7 μM final concentration), 0.125 μL of BSA (0.25 μg μL^-1^ final concentration) and 7.875 μL of ultra-pure sterile water. qPCR reactions consisted of an initial denaturation step at 94°C for 15 min, followed by 40 cycles of denaturation at 95°C for 15 s, annealing for 30 s (54°C for bacterial and *Dehalogenimonas* 16S rRNA genes and 59°C, for *Dehalococcoides* 16S rRNA gene, S1 Table) and elongation at 72°C for 30 s. Standard curves were generated from serial dilutions (10^1^ to 10^8^ copies per reaction) of plasmids containing 16S rRNA synthetic genes for each DEH phylotype synthesized by Biomatik Corporation (Cambridge, Ontario, Canada) and included in each run in triplicate.

### Metagenome annotation

The metagenomic dataset (NCBI accession number SRA049219) used in this study corresponded to a gDNA sample collected at 90 m depth in 2011 in Lake Pavin by Denonfoux *et al*. [39]. It included 116,365 high quality reads with a total of 54,740,219 bp. The length of sequence reads ranged from 335 to 598 bp (mean size ~471 bp). Read assembly was done with Roche’s GS *de novo* Assembler (‘Newbler’) v2.6 and required read overlaps of ≥60 bp at ≥95% nucleotide identity. After assembly, the contigs (maximum length 8,485 bp) and the 99,917 non-assembled reads were analyzed and annotated using the MG-RAST server (MG-RAST ID: 4487833.3) [40] to identify candidate genes corresponding to *rdhA* and insertion element (IS) sequences. Candidate genes identified in the metagenomic dataset were amplified by PCR from gDNA samples using specific primer sets (S1 Table) and confirmed by cloning and sequencing as described above. Protein encoding sequences recovered in this study are available in GenBank (accession numbers KJ475645 to KJ475665).

### Solution hybrid selection capture of gDNA fragments

Solution hybrid selection capture was performed on the gDNA sample collected at 68 m depth on the basis of the phylogenetic analyses and the qPCR results. In total, 21 capture probes ranging from 40 to 56 bp were manually designed (S2 Table). Adaptors were added at each end, resulting in 70 to 86-mer hybrid probes consisting of 5’-ATCGCACCAGCGTGT-X_40-56_-CACTGCGGCTCCTCA-3’ to allow double-stranded DNA probe production by PCR amplification using primers T7-A and B (S2 Table). The probes were purchased from Eurogentec (Seraing, Belgium). Biotinylated RNA baits were prepared from double-stranded DNA probes, as previously described [41]. The library preparation and hybridization capture were conducted as described by Denonfoux *et al*. [39] using Roche’s GS FLX Titanium General Library Preparation kit (Roche, Branford, CT, USA). Briefly, libraries were constructed from 10 μg of gDNA obtained from 3 L water samples and sheared by nebulization for 5–10 s under 0.2 Pa. The DNA fragments were size selected with Ampure beads (Beckman Coulter Genomics, Danvers, MA, USA). After purification, fragment end polishing, adaptor ligation (A and B adaptor keys) and fill-in reactions, the libraries, containing DNA fragments up to 5 kb, were PCR amplified with 454-TiA and 454-TiB primers (S2 Table). After capture and increase of the DNA yield by PCR amplification with 454-TiA and 454-TiB primers, the PCR products were cloned using the TOPO TA cloning kit. Plasmids were screened for high-size inserts by digestion with EcoRI. Positive clones were sequenced using the Sanger sequencing technology by the Eurofins Genomics sequencing services (https://eurofinsgenomics.eu). Sequences were processed and joined using the Staden Package [42]. Primer sequences were removed from paired-end consensus sequences. For inserts above 1,500 bp, full-length sequences were obtained using internal primers. Sequence data are available in GenBank under accession numbers KJ475550 to KJ475585.

## Results

### Lake Pavin water column stratification

**Fig 1:**
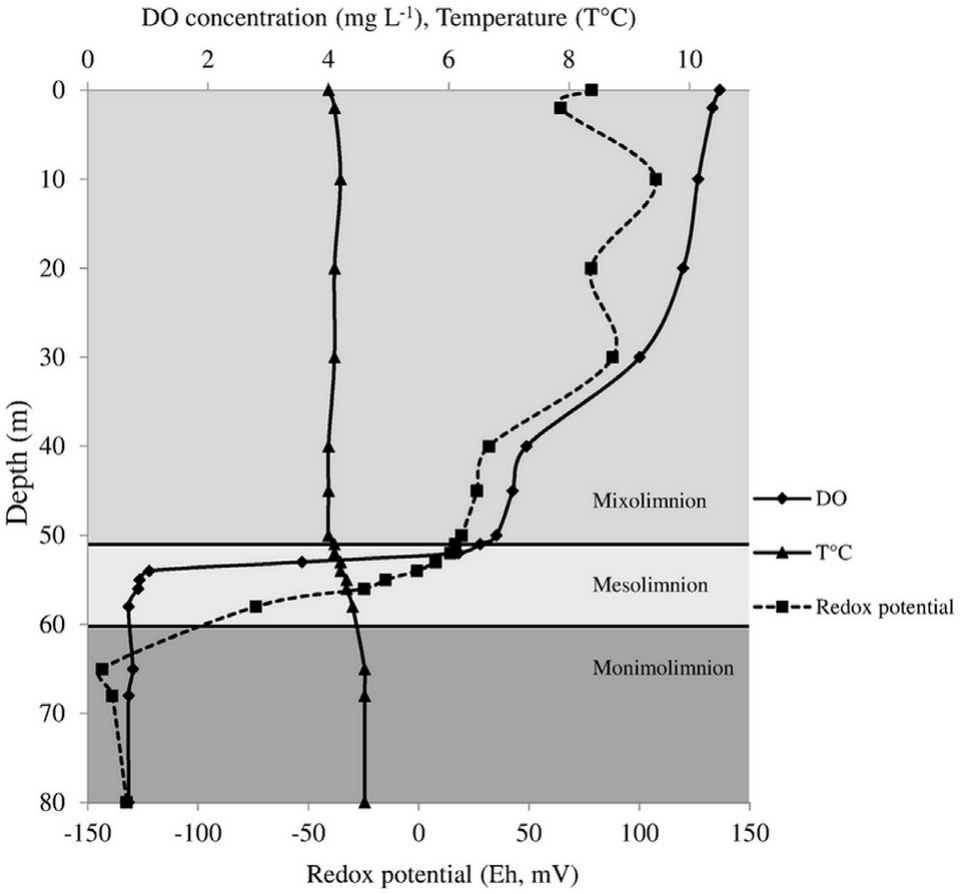
Physical parameter measurement along Lake Pavin water column. Vertical profiles of dissolved oxygen (DO) and temperature measured in March 2012 using the submersible YSI GRANT 3800 multiparameter probe. Redox potential (Eh) measured in June 2013 in reference to a standard electrode and expressed in millivolts (mV) (Didier Jézéquel personal communication).

In March 2012, when samples were taken, the mixolimnion of Lake Pavin was measured from 0 to ~53 m, the mesolimnion ~53 to ~60 m and the monimolimnion ~60 m to the bottom of the lake at 92 m (Fig 1). Temporal variations of the vertical mesolimnion’s depth have already been described [43]. They were interpreted as variations in the intensity and duration of the annual circulation of the mixolimnion depending on meteorological conditions at the surface of the lake. Within the first meters of the mesolimnion, the oxygen concentration decreased sharply to become undetectable at the bottom of the layer. In this study, four water samples were collected in the top of the anoxic zone covering the lower part of the mesolimnion (58 m) and the monimolimnion (60 m, 63 m and 68 m) (Fig 1). Because of the rapid changes in physico-chemical conditions at these depths, this sampling strategy aimed at covering most of microbial populations growing in this zone. Two additional samples were collected in the deeper part of the monimolimnion (80 m; deep anoxic zone) and in the lower part of the mixolimnion (52 m; 6.2 mg L^-1^ O_2_; oxic zone) in order to provide comparative information. As shown by the redox potential values, the deep anoxic layer of Lake Pavin was highly reducing (Fig 1).

### Microbial population analysis

**Fig 2:**
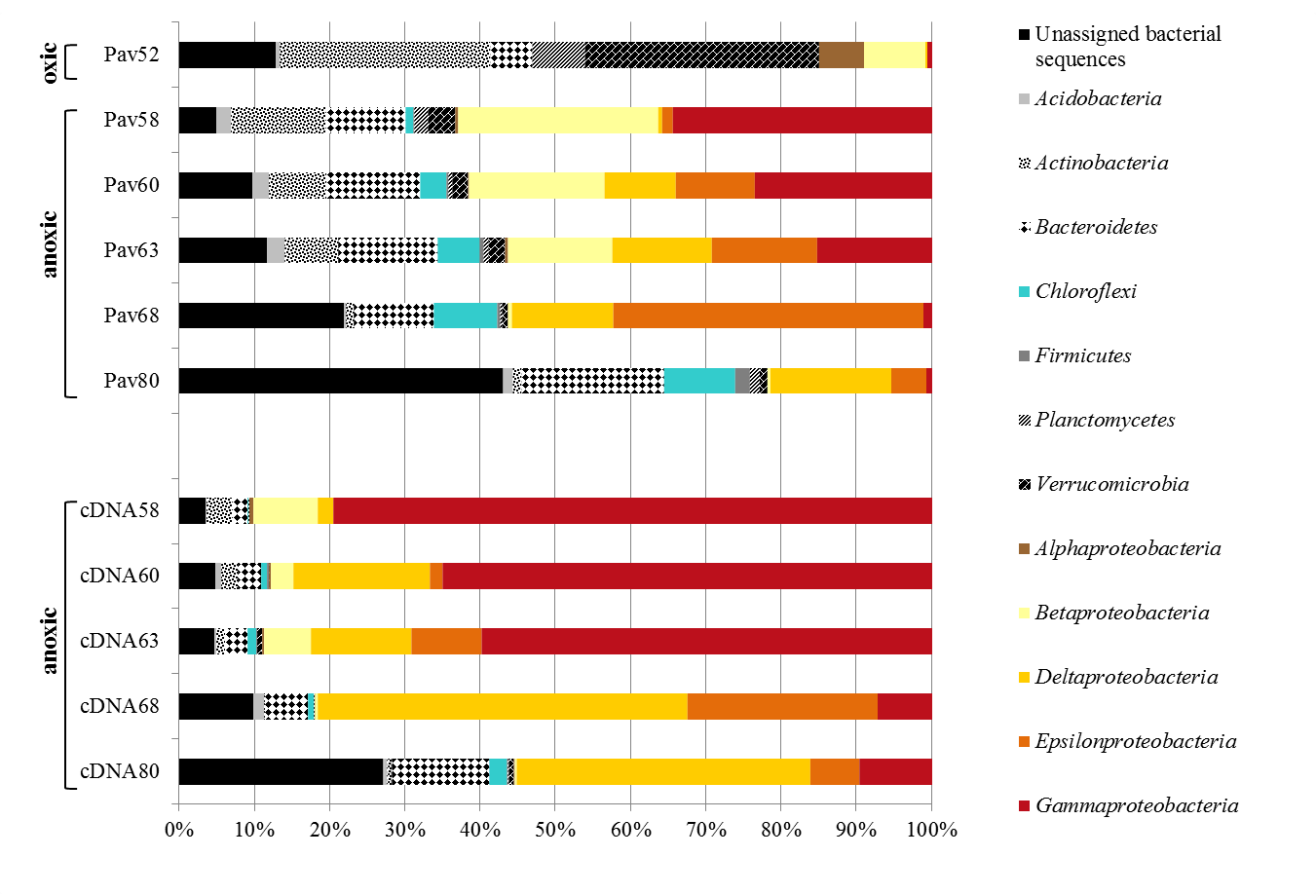
Vertical distribution and relative abundance of the main bacterial phyla. Sample names starting with “Pav” indicate the samples were amplified from gDNA, names starting with “cDNA” indicate that the samples were processed by reverse transcription from mRNA. The number in the name indicates sample depth.

Small subunit ribosomal RNA (16S rRNA) gene sequence analysis was used at the selected depths using both universal and specific (DEH, *Sulfurospirillum*, *Dehalobacter*, *Desulfitobacterium* and *Geobacter*) primers (S1 Table). Pyrosequencing with universal primers generated a total of 27,754 high quality bacterial reads from the six gDNA samples and the five cDNA samples (S3 Table). After read clustering and singleton removal, a total of 994 OTUs (26,889 reads) were affiliated with 18 phyla and 8 candidate divisions. Distinct bacterial communities were evident in the oxic and anoxic zones at the phylum level (Fig 2). For instance, the *Actinobacteria* and *Verrucomicrobia* that accounted for up to 59% of the sequences in the oxic zone were much less abundant in the anoxic zone. In the anoxic layer below a depth of 52 m, *Proteobacteria* abundance increased to about 60% of the sequences, whereas they represented only 15% of the sequences in the oxic layer. At a finer taxonomic resolution, *Gamma-Proteobacteria* (mostly affiliated with the methanotrophic *Methylococcales*) and *Beta-Proteobacteria* (mostly affiliated with *Rhodocyclales*, *Burkholderiales* and *Methylophilales*) dominated the upper waters (from 58 m to ~63 m depth), whereas the bottom waters (below 63 m depth) were dominated by *Delta-Proteobacteria* (mostly affiliated with *Desulfobacterales* and *Syntrophobacterales*) and *Epsilon-proteobacteria* (affiliated with *Campylobacterales*). Moreover, cDNA samples were largely dominated by 16S rRNA sequences from known aerobic methanotrophs, which represented up to 80% of sequences at 58 m depth. These results are consistent with observations in Lake di Cadagno (Switzerland) where methane consumption just below the oxycline was due to direct coupling of aerobic methane oxidation to oxygenic photosynthesis [44]. A depth-specific trend was also observed for the *Chloroflexi*, which were not detected in the oxic zone but increased in abundance with depth in the anoxic zone to an abundance of about 10% at 80 m depth. These sequences were mostly affiliated with *Anaerolineales* and the GIF9 class.

**Fig 3:**
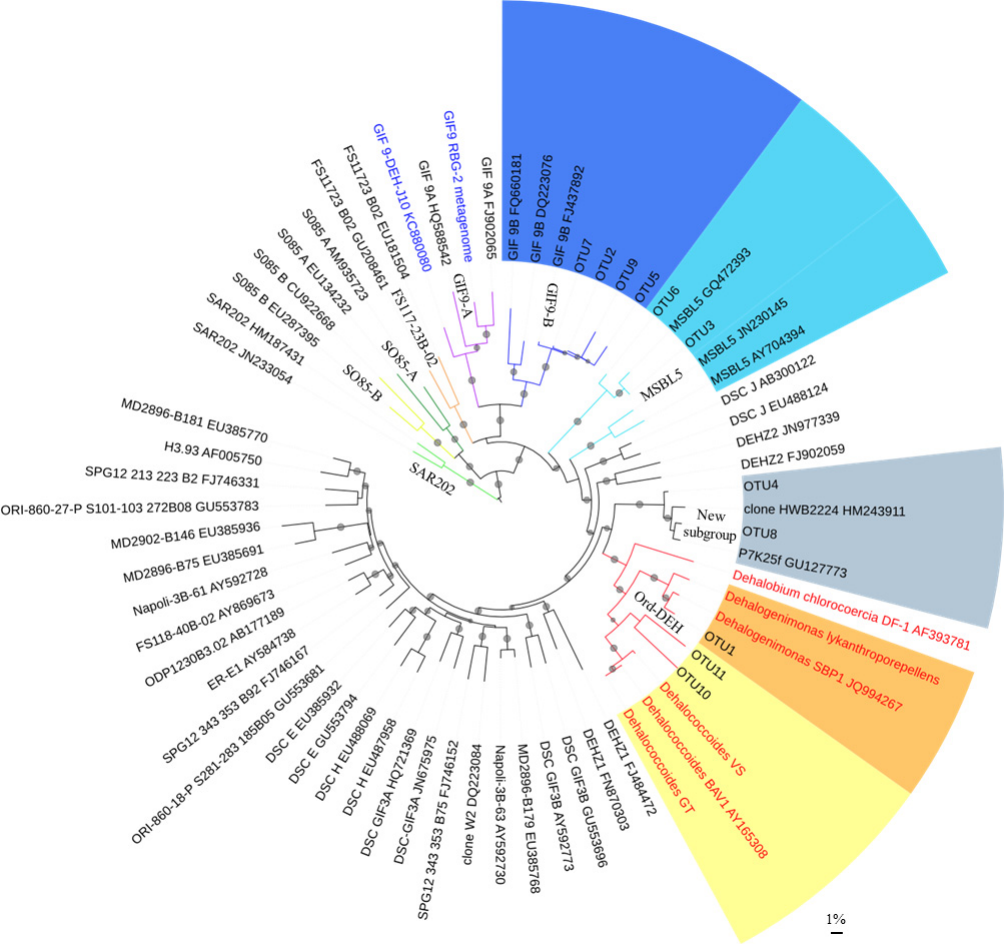
Phylogenetic tree of *Dehalococcoidia* (DEH) 16S rRNA gene sequences highlighting the phylogenetic position of the 11 OTUs obtained from Lake Pavin water column. The phylogenetic analysis was performed using the neighbor-joining method; branches with bootstrap values (1000 replicates) of at least 50% are marked with a dot. The tree was constructed using MEGA version 6.0 [38]. The branches were coloured using iTOL tool [45]. Sequences from this study fall into three classes defined by Wasmund *et al*. [15]: GIF-9B in dark blue, MSBL5 in light blue and Ord-DEH in orange (*Dehalogenimonas*) and in yellow (*Dehalococcoides*). An additional class not previously described is presented in grey as an annotated new subgroup. Sequences of cultured DEH are highlighted in red. Sequences derived from single-cell genome DEH-J10 [16] and an aquifer sediment metagenome-derived genome RBG-2 are highlighted in blue. The scale bar represents 1% sequence divergence.

In parallel to pyrosequencing, we sequenced a total of 65 nearly complete 16S rRNA gene sequences *via* Sanger sequencing using primers targeting specific groups of OHRB. Of these, 44 were affiliated with DEH, eight with *Sulfurospirillum* and 13 with *Geobacter*. Together, Sanger and pyrosequencing revealed the presence of 20 different OTUs closely related to known OHRB (Table 1). Of these, 11 were closely related to obligate OHRB (*Dehalogenimonas* and *Dehalococcoides*) and nine to facultative OHRB (*Sulfurospirillum*, *Geobacter* and *Desulfomonile*). All DEH OTUs were found in anoxic layers, with the exception of OTU5, which contained one sequence amplified from DNA collected at 52 m depth. OTU1 exhibited 98% nucleotide identity with *Dehalogenimonas alkenigignens* SBP1, whereas OTU2 to 11 were more divergent (80 to 91% nucleotide identity with known DEH strains; Table 1). Of the 11 DEH-related phylotypes, nine fell in three subgroups defined by Wasmund *et al*. [15], i.e. four in GIF-9B, three in Ord-DEH (which includes all known DEH strains) and two in MSBL5 (Fig 3). The remaining two OTUs (OTU4 and 8) clustered in a new subgroup. Among the OTUs close to facultative OHRB, four were 93–96% identical with 16S rRNA genes of members of the *Geobacter* genus, four were 90–93% identical with members of the *Desulfomonile* genus, and one had 99% identity with *Sulfurospirillum multivorans* (Table 1).

**Table 1:** Overview of sequences from Lake Pavin related to known OHRB.

OTU^a^	No. of Sanger clones		No. of 454 FLX reads		Closest hit in the database			OHRB type
	gDNA	cDNA	gDNA	cDNA	Organism	Accession no.	% id	
OTU1	22	3	2	0	*Dehalogenimonas alkenigignens* SBP1	JQ994267	98	Obligate OHRB
OTU2	1	5	270	27	*Dehalococcoides mccartyi* BTF08	NR_102515	86	Obligate OHRB
OTU3	1	7	6	9	*Dehalogenimonas lykanthroporepellens* BL-DC-9	NR_044550	85	Obligate OHRB
OTU4	3	0	6	2	*Dehalogenimonas alkenigignens* SBP1	JQ994267	88	Obligate OHRB
OTU5	1	0	0	0	*Dehalococcoides mccartyi* BTF08	NR_102515	84	Obligate OHRB
OTU6	0	1	0	0	*Dehalogenimonas lykanthroporepellens* BL-DC-9	NR_044550	85	Obligate OHRB
OTU7	0	0	9	1	*Dehalococcoides mccartyi* BTF08	NR_102515	80	Obligate OHRB
OTU8	0	0	4	0	*Dehalococcoides mccartyi* BTF08	NR_102515	84	Obligate OHRB
OTU9	0	0	6	1	*Dehalococcoides mccartyi* BTF08	NR_102515	80	Obligate OHRB
OTU10	0	0	0	5	*Dehalococcoides mccartyi* VS	NR_074326	89	Obligate OHRB
OTU11	0	0	0	2	*Dehalococcoides mccartyi* VS	NR_074326	91	Obligate OHRB
OTU12	8	nd	0	0	*Sulfurospirillum multivorans*	NR_044868	99	Facultative OHRB
OTU13	13	nd	3	11	*Geobacter urniireducens*	NR_074940	96	Facultative OHRB
OTU14	0	nd	3	12	*Geobacter psychrophilus*	NR_043075	98	Facultative OHRB
OTU15	0	nd	0	4	*Geobacter psychrophilus*	NR_043075	97	Facultative OHRB
OTU16	0	nd	0	2	*Geobacter pelophilus*	NR_026077	93	Facultative OHRB
OTU17	nd	nd	6	144	*Desulfomonile tiedjei*	CP003360	93	Facultative OHRB
OTU18	nd	nd	0	5	*Desulfomonile tiedjei*	CP003360	91	Facultative OHRB
OTU19	nd	nd	0	1	*Desulfomonile tiedjei*	CP003360	90	Facultative OHRB
OTU20	nd	nd	0	9	*Desulfomonile limimaris*	AF282177	93	Facultative OHRB

^a^The OTUs were defined at a threshold of 97%.

nd, not determined; % id, identity percent; no., number.

**Fig 4:**
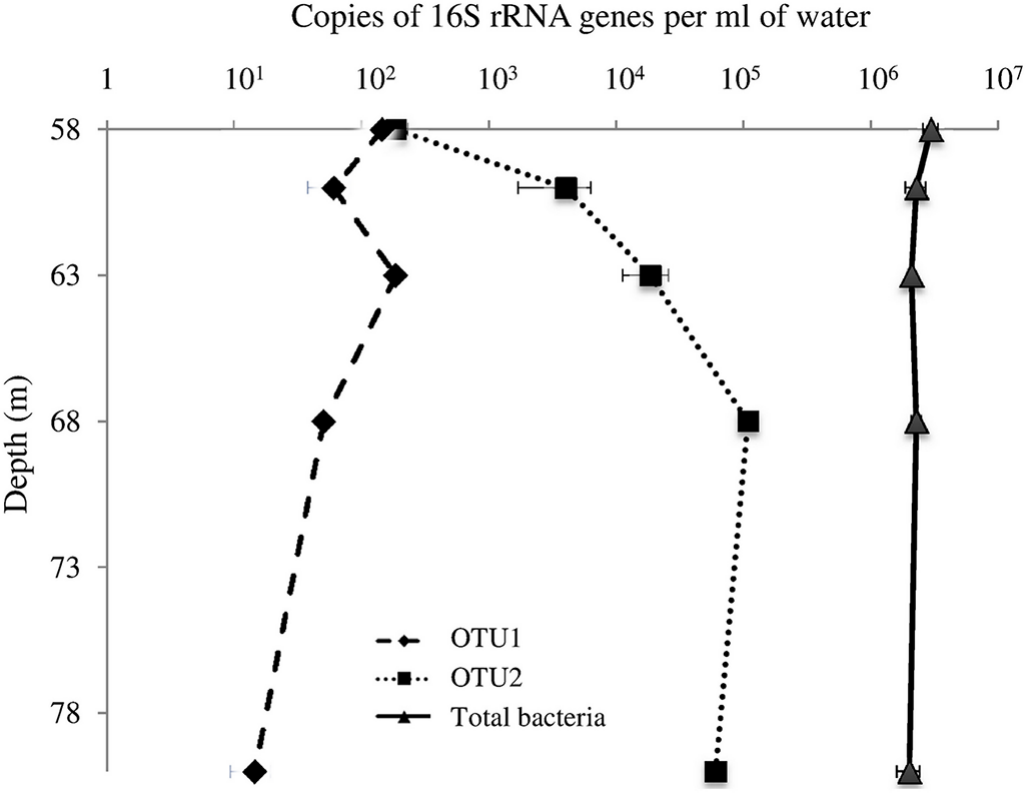
Quantification of ‘the total density of’ bacterial 16S rRNA genes and of two DEH-related phylotypes in Lake Pavin water column. Bacteria are represented by filled triangles. For DEH-related phylotypes: OTU1 is represented by filled diamonds and a dashed line and OTU2, by filled squares and a dotted line. Shown are mean values and standard deviations of triplicate PCR reactions.

We then assessed the density of the two most abundant DEH OTUs along the water column by qPCR (Fig 4), i.e. OTU1, which was closely related to *Dehalogenimonas alkenigignens* SBP1 and most often retrieved by the Sanger sequencing approach, and OTU2, which was closely related to *Dehalococcoides* sequences and the most abundant DEH sequence in the pyrosequencing dataset (Table 1). OTU1 was detectable at only low abundance (approximately 10^2^ copies mL^-1^) along the water column and total density decreased with water depth. In contrast, a 10^3^-fold increase in OTU2 copy numbers was observed between depths of 58 m (approximately 10^2^ copies mL^-1^) and 68 m (10^5^ copies mL^-1^), and slightly decline at 80 m (6 x 10^4^ copies mL^-1^). The concentration of total bacterial 16S rRNA genes was >2 x 10^6^ copies mL^-1^ at all analyzed depths. Overall, OTU2 may represent more than 3% of the total 16S rRNA gene copies in the deepest sample location. These results corroborate the results from the population analysis that DEH occurred preferentially in the deep anoxic layers of Lake Pavin.

### Application of gene capture for enrichment of DEH genomic fragments

In order to obtain more genomic information from DEH in Lake Pavin, we used an enrichment strategy for specific gDNA fragments using targeted hybridization probes and applied it to the enrichment of DEH gDNA fragments from the water sample collected at 68 m depth where most DEH OTUs were found by pyrosequencing. We used a two-step capturing approach: in the first step (“capture 1”), we used probes targeting DEH genes identified through the PCR product sequencing (i.e., 16S rRNA amplicons) and the exploration of the metagenome dataset (i.e., *rdhA* genes and insertion sequence (IS) elements because of their frequent link to *rdhA* genes in sequenced DEH genomes) [46]. In a second step (“capture 2”), we used the information of genomic fragments captured during the first step to design fragment-specific probes and enriched more fragments from a targeted locus.

### Capturing DEH gDNA fragments by using 16S rRNA genes as target

**Fig 5:**
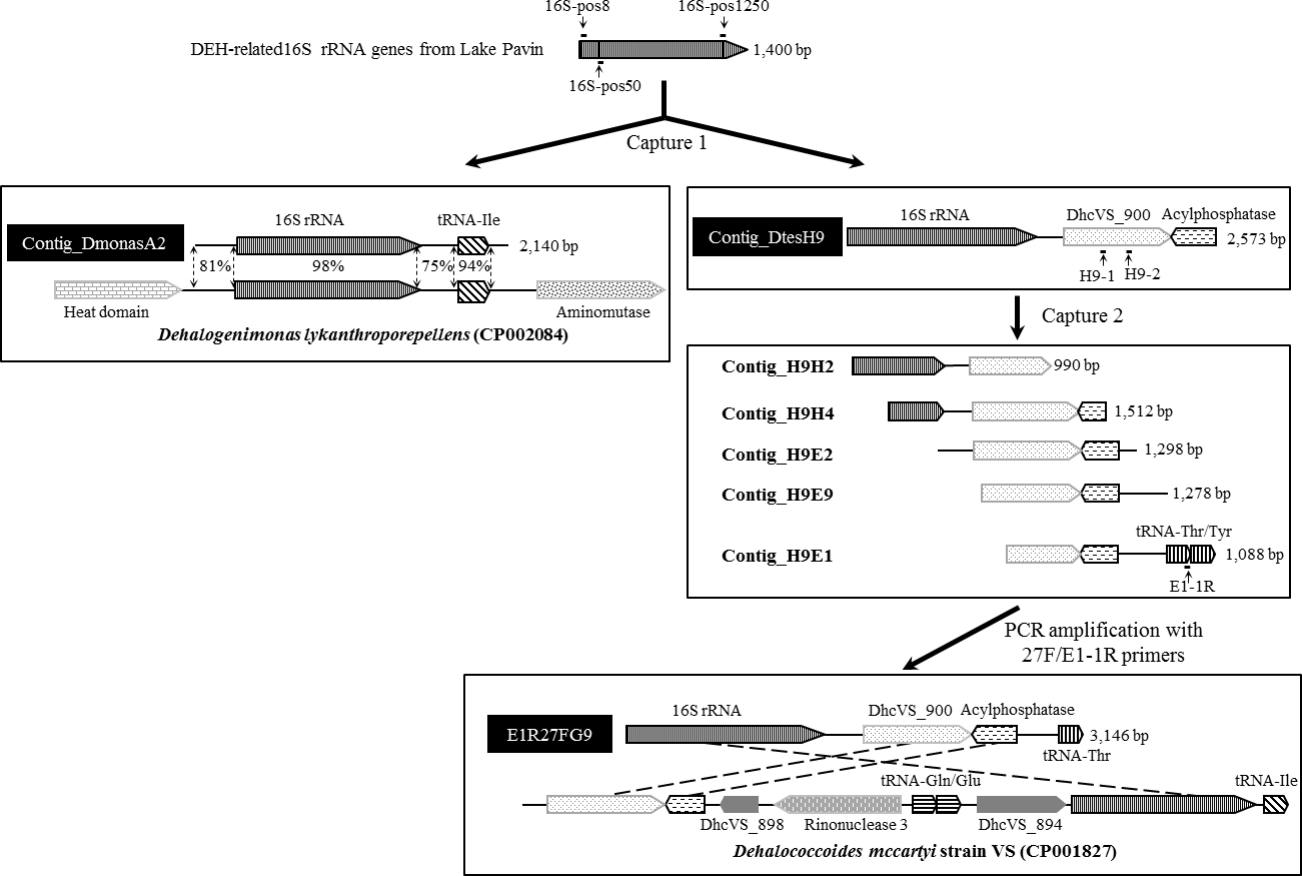
Genomic fragments obtained from Lake Pavin using the specific-gene capture approach targeting DEH 16S rRNA genes. The genomic fragments were recovered at 68 m depth by specific-gene capture approach using probes targeting the 16S rRNA genes of DEH phylotypes (first capture experiment) and a hypothetical protein (second capture experiment) identified in Lake Pavin water column. 16S-pos8, 16S-pos50, 16S-pos1250, H9-1 and H9-2: capture probes; E1-1R: PCR primer.

The rRNA genes of fully or nearly-fully sequenced DEH are not linked in the typical arrangements for bacteria, i.e., they are not organized into operons and the 16S rRNA gene is located hundreds of kbp away from the 23S rRNA gene [47]. To study the organization of the rRNA genes within the DEH-related strains identified in Lake Pavin, we designed three capture probes targeting different positions of the 16S rRNA genes (16S-pos8, 16S-pos50 and 16S-pos1250) (Fig 5, S2 Table). Then we used them to isolate larger genomic fragments containing both the 16S rRNA genes and their neighboring genes. Of the 57 genomic fragments randomly sequenced after the first capture experiment, one with a size of 2,140 bp (Contig_DmonasA2) contained a 16S rRNA gene closely related to OTU1 (99.4% identity) and an isoleucine tRNA (Ile tRNA) gene (Fig 5); both genes showed highest similarity with genes of *Dehalogenimonas* strains (98 and 94% identity, respectively) (S4 Table). Intergenic sequences flanking the 16S rRNA sequence also showed the highest identity with those flanking the 16S rRNA gene of *D*. *lykanthroporepellens* (81% and 75% identity with the 5’ and 3’ intergenic sequences, respectively, Fig 5). The “capture 2” attempts to isolate larger genomic fragments were unsuccessful using probes designed from the Contig_DmonasA2 sequence.

A second genomic fragment with a size of 2,573 bp containing a 16S rRNA gene very similar to OTU2 (98.5% identity) (Contig_DtesH9) was also recovered after the first capture (Fig 5). To improve our data on this contig, a second capture experiment was performed using two novel capture probes (H9-1 and H9-2) derived from the retrieved sequence together with the initial library constructed from the 68 m sample. This allowed the isolation of five additional genomic fragments ranging in size from 990 to 1,512 bp. Their assembly (threshold at 98% identity) resulted in a consensus sequence of 3,178 bp including the nearly complete 16S rRNA gene, a gene encoding a protein annotated as hypothetical (most similar to DhcVS_900), a gene encoding an acylphosphatase, as well as threonine and tyrosine tRNA genes (Fig 5). The closest homologs for the hypothetical protein and the tRNAs were found among DEH strains (S4 Table). We confirmed the existence of this genomic fragment at 68 m depth in Lake Pavin by PCR amplification using the 27F universal 16S rRNA gene primer and a reverse primer (E1-1R, S1 Table) targeting the threonine tRNA gene (amplicon E1R27FG9, Fig 5). Three of the genes detected in this contig (genes for 16S rRNA, the hypothetical protein and acylphosphatase) were also found at the same locus in most *Dehalococcoides* genomes, but with a different organization. Overall, our results showed that 16S and 23S rRNA genes are physically distant in the genome of Lake Pavin phylotypes represented by OTU1 and OTU2. Hence, those strains seem to have rRNA gene features as outlined in previously sequenced DEH.

### Capturing DEH gDNA fragments using *rdhA* gene probes

**Fig 6:**
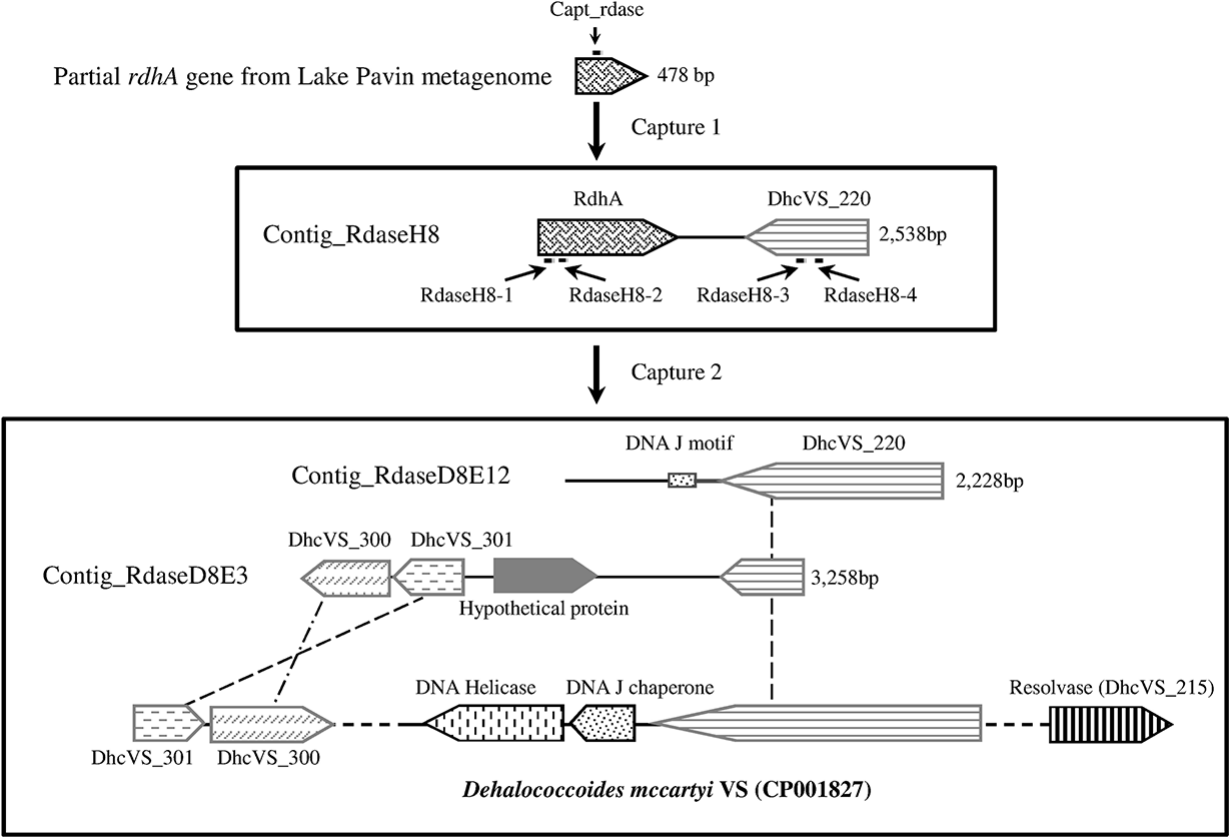
Genomic fragments obtained using the specific-gene capture approach targeting an *rdhA* sequence identified in a Lake Pavin metagenome [39]. The genomic fragments were recovered from the 68 m-depth sample by specific-gene capture approach using probes targeting a *rdhA* gene (first and second capture experiments) and a hypothetical protein (second capture experiment) identified in Lake Pavin water column. Capt_rdase, RdaseH8-1, RdaseH8-2, RdaseH8-3 and RdaseH8-4: capture probes.

Chromosomes of cultivated and sequenced DEH strains carry 10 to 36 different *rdhA* genes [46–48]. These *rdhA* genes are not strongly conserved making it difficult to define broad capture probes [49]. In this context, one of the strategies adopted to isolate *rdhA* genes from Lake Pavin was based on the exploration of a previously described metagenomic dataset (MG-RAST ID: 4487833.3) [39] to detect *rdhA* signatures followed by specific sequence enrichment using our gene capture method. Only one *rdhA* homologous sequence (478 bp) was detected in the metagenomic dataset. It encoded a protein with 62% amino acid identity with the tetrachloroethene reductive dehalogenase (PceA) of *Heliobacterium modesticaldum* Ice1 (YP_001680129). The most similar *rdhA* gene in DEH had 50% protein identity over a stretch of 80 amino acids. We confirmed the presence of this sequence in Lake Pavin by PCR amplification and then used it to design a specific capture probe (Capt_rdase, Fig 6, S2 Table).

After capture, we sequenced thirty clones. One contig (Contig_RdaseH8) with a size of 2,538 bp contained a nearly complete sequence of the targeted *rdhA* (1,056 bp, 55% protein identity across 333 amino acids with *H*. *modesticaldum* PceA) and a second nearly complete gene encoding a hypothetical protein (most similar to DhcVS_220, acc. no. ACZ61382) (Fig 6). This second protein was ∼62% identical along an alignment of 300 amino acids with a protein only found in *Dehalococcoides* (S4 Table). A second capture experiment using four additional probes (RdaseH8-1, RdaseH8-2, RdaseH8-3 and RdaseH8-4, Fig 6, S2 Table) provided two additional fragments of 2,228 (RdaseD8E12) and 3,258 bp (RdaseD8E3). These fragments contained sequences with high nucleotide identity with the gene DhcVS_220 but not to the targeted *rdhA* gene (S4 Table). The largest fragment harbored three genes encoding hypothetical proteins. Two of them (most similar to DhcVS_300, acc. no. ACZ61461, and DhcVS_301, acc. no. ACZ61462) were commonly found in most DEH strains (48–50% and 56–63% protein identity, respectively) (Fig 6) but not in any other bacterial genomes.

### Capturing DEH gDNA fragments by using probes targeting IS elements

The other capture strategy adopted to isolate supplemental *rdhA* genes from Lake Pavin was to target IS elements. Indeed, all DEH strains currently sequenced carry a large number of IS elements in their chromosome and most of the *rdhA* genes are located in the neighborhood of these elements. For example, the *D*. *lykanthroporepellens* genome contains 74 IS elements [46] and 19 *rdhA* genes, of which 75% are adjacent to an IS element [7]. Assuming that the *Dehalogenimonas*-related phylotype (OTU1) detected in Lake Pavin has a similar gene organization, all *D*. *lykanthroporepellens* IS protein sequences were downloaded from the NCBI database and used to screen the metagenomic dataset using TBLASTn (minimum 30% protein identity over a stretch of 100 amino acids). Three of 16 IS sequences identified in the metagenome had highest similarity with different regions of the IS911 element of *D*. *lykanthroporepellens* (74–75% protein identity over the whole length), a member of the IS3 family composed of two consecutive overlapping ORFs (*orfA* and *orfB*). The presence of these sequences in Lake Pavin was confirmed by PCR amplification. The sequences were used to design nine further specific capture probes (S2 Table).

**Fig 7:**
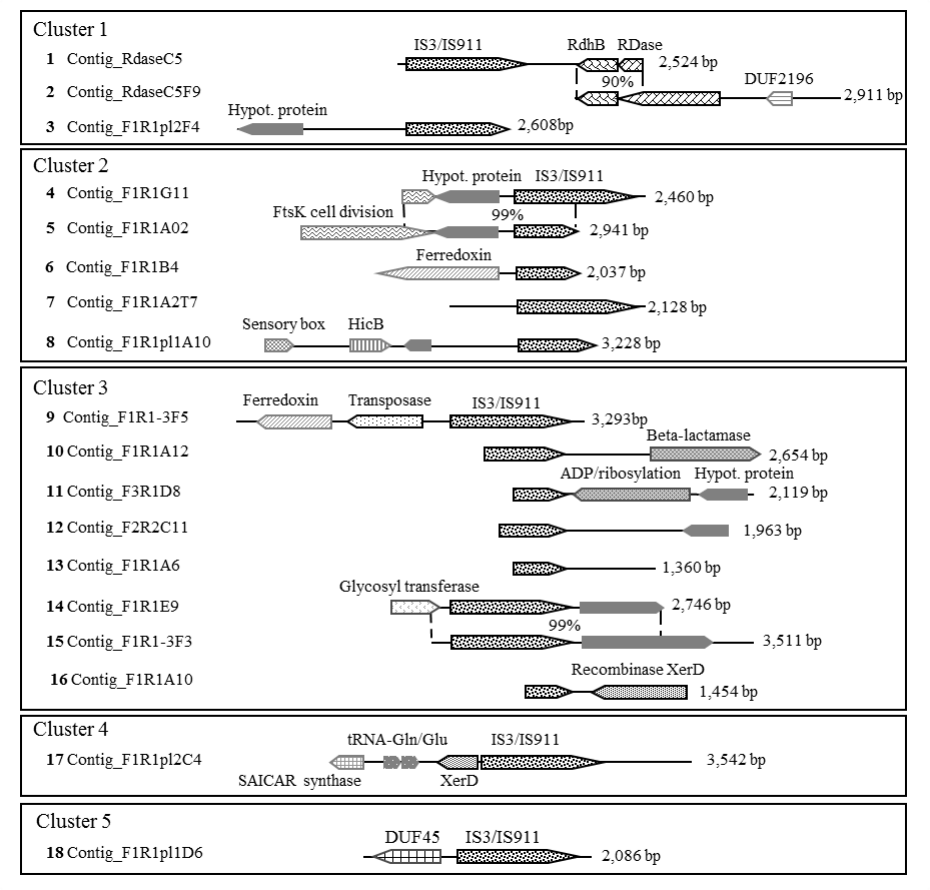
Genomic fragments obtained from Lake Pavin using the specific-gene capture approach targeting insertion sequences. The genomic fragments were recovered at 68 m depth using the capture approach targeting IS911, an insertion sequence (IS) belonging to the IS3 family. In total, 18 genomic fragments with IS are grouped together into five distinct clusters.

Because the 11 copies of IS911 present in the *D*. *lykanthroporepellens* chromosome (CP002084) had high sequence conservation (98–100% nucleotide identity), we hypothesized that IS911-containing genomic fragments recovered from Lake Pavin having an IS with at least 98% sequence identity could be carried by a single microbial population. Hence, we decided to use this threshold to cluster captured sequences. Of the 31 clusters obtained from almost 200 sequenced clones, five harbored at least one ORF that best matched with a DEH-related sequence in addition to the IS, and were thus further analyzed. These five clusters represent 18 individual DNA fragments (ranging from 1,360 to 3,542 bp) for a total of 45,565 bp. Their analysis allowed the identification of 59 ORFs: 30 encoding IS911 elements, 19 annotated as various functional genes and 10 as hypothetical (Fig 7, S5 Table). Of the 19 functional genes, one encoded a protein showing 47–52% identity with an anchoring protein (RdhB) of DEH strains and one another, 59% identity with the RDase of an uncultured bacteria originating from marine sediment (BAI47792). These two genes, organized in an operon, were present on the Contig_RdaseC5 fragment (2,524 bp) in Cluster 1 (Fig 7). The closest relatives of this RDase in DEH strains showed only 37–39% identity over a length of 58 to 62 amino acids.

In order to obtain the full length of the *rdhA* gene, a second capture experiment using two newly designed capture probes (C5_1 and C5_2, S2 Table) was performed as previously described, which allowed the isolation of an additional genomic fragment (Contig_RdaseC5F9) of 2,911 bp (Fig 7). This fragment contained both full-length sequences of the *rdhA* and *rdhB* genes as well as a gene encoding a hypothetical protein with no homolog among DEH (S5 Table). The overlapping stretch between the fragments Contig_RdaseC5 and Contig_RdaseC5F9 (540 bp long) was 90% identical. It should be noticed that the RDase sequence identified in the Contig_RdaseC5F9 (Fig 7) shared only 18% protein identity with the RDase sequence identified in the Contig_RdaseH8 using the capture approach targeting a *rdhA* gene (Fig 6).

## Discussion

Disparate bacterial communities were detected between oxic and anoxic parts of the water column in Lake Pavin, consistent with patterns observed in other meromictic lake ecosystems [21,22,50]. Moreover, diversity indices (Chao1, Abundance-based coverage estimator (ACE) and Shannon, S3 Table) revealed that richness was highest in the monimolimnion similar to what has been observed in the monimolimnion of other stratified lakes [21,44,51]. With the description of 20 OTUs closely related to known OHRB, our results also demonstrate that several OHR candidate phyla are present in Lake Pavin monimolimnion, including facultative (*Desulfomonile*, *Geobacter* and *Sulfurospirillum*) and obligate (*Dehalogenimonas* and *Dehalococcoides*) OHRB populations. Such OHRB diversity was yet-unknown in uncontaminated lake water column, as previous related work has focused mainly on soil [11], river [52] and sedimentary habitats of freshwater lakes and marine systems [16–19,53], but not on water column. Overall, our results differ from previous studies conducted either on contaminated [54–56] or on uncontaminated systems [20,57,58] where *Dehalobacter* (obligate OHRB) and *Desulfitobacterium* (facultative OHRB) genera within the *Firmicutes* were frequently detected in association with DEH, which is not the case in Lake Pavin. In addition, we report the occurrence of *Dehalogenimonas* relatives in pristine environment; previous studies have detected members of this genus mainly in contaminated samples [46,56,59,60].

DEH are the only obligate OHRB identified in the present work. Most of them were detected as both present and active using DNA and RNA results, respectively. With the exception of OTU1 which is 98% similar to the reference strain *D*. *alkenigignens* SBP1, all other DEH-related phylotypes identified in this study have relatively low sequence identity with cultivated strains. The best matches using BLASTn searches were with uncultured bacteria found in various ecosystems, mostly lake sediments [61,62]. Unlike classical 16S rRNA gene sequencing approaches, the powerful gene capture method used in this study allowed the sequencing of complete 16S rRNA genes and their neighboring sequences for two of the 11 DEH-related phylotypes (OTU1 and OTU2). *In silico* analysis confirms their rRNA genes are not arranged in the standard operon structure (16S-23S-5S). In OTU1 phylotype, we found a gene organization pattern that is identical to that observed in all of the completely sequenced DEH strains (*Dehalococcoides mccartyi* strains 195 (CP000027), VS (CP001827), BAV1 (CP000688), CBDB1 (AJ965256), DCMB5 (CP004079), BTF08 (CP004080), GT (CP001924), GY50 (CP006730), CG1 (CP006949), *Dehalogenimonas lykanthroporepellens* (CP002084) and *Dehalogenimonas* sp WBC-2 (CP011392) and known to perform OHR, i.e., the gene encoding an Ile tRNA gene is located immediately downstream of the gene coding for 16S rRNA [47]. In contrast, in OTU2 phylotype as well as in partially sequenced RBG-2 [19] and DEH-J10[16] strains, genes other than the Ile tRNA gene are located downstream of the 16S rRNA gene.

Quantitative PCR allowed us to reveal a depth-specific trend for the presence of OTU2, which increases in abundance between 58 m and 68 m depth. This trend was not observed for the *Dehalogenimonas*-related phylotype represented by OTU1. According to the 16S rRNA gene phylogeny, OTU2 belongs to the GIF9 class. This class, without cultivated representatives, has been identified in many environments from methane seeps to bioreactors [19,21,53,63]. For example, a recent study conducted on the meromictic Lake Sakinaw (Canada) showed that the GIF9 class and *Syntrophobacterales* including the genera *Smithella* and *Syntrophus* were abundant in the methanogenic bottom layer of the lake [21]. Such abundance and co-occurrence were also observed in Lake Pavin with similar methanogenic conditions [31,32]. Currently, two nearly complete genomes are available for this group, one obtained from an aquifer sediment using metagenomics (RBG-2, [19]) and the other from marine sediment by using single-cell genomics (DEH-J10 [16]). On the basis of genomic analysis and the lack of *rdhA* genes in their chromosome, both strains are supposed to use other processes than OHR for energy conservation. OTU2 shares only 85.5% 16S rRNA gene sequence identity with the 16S rRNA gene of RBG-2 and 86.5% with that of DEH-J10, indicating that we detected a distinct phylotype among this group. The closest 16S rRNA gene sequence found in public databases was annotated as an uncultured bacterium derived from the anoxic area of Green Lake, a meromictic lake in New York (accession number FJ437868, 98% nucleotide identity).

The detection of DEH-related 16S rRNA genes in an ecosystem, especially when they are divergent from reference strains, is not sufficient to conclude that they are able to respire with organohalides. In contrast the detection of *rdhA* genes, the key genes in OHR, is more indicative for OHR potential. The *rdhA* gene family exhibits significant diversity across gene sequences. The capture approach applied in this study is a powerful PCR-independent tool to investigate previously uncharacterized *rdhA* genes. It enabled the identification of two novel *rdhA* genes, one using probes targeting a *rdhA* gene sequence identified in a metagenomic dataset from Lake Pavin (RdaseH8) and the second, using probes targeting adjacent IS elements (Contig_RdaseC5 and Contig_RdaseC5F9). The first RDase shows highest similarity with *H*. *modesticaldum* PceA (YP_001680129) whereas the second was more similar to an uncharacterized *rdhA* gene detected in Pacific Ocean sediments (BAI47792). The capture approach also gave sequence information from the direct *rdhA* genomic neighborhoods. Only this enabled the reliable classification of the genomic fragments and their encoded *rdhA* genes to genomes of DEH. The *rdhA* gene carried by Contig_RdaseC5F9 has a TAT motif in its sequence, is organized in an *rdhAB* operon and associated with an IS element. This organization is common for most *rdhA* genes occurring in DEH genomes. For instance, 14 of the 19 *rdhA* genes present in the *D*. *lykanthroporepellens* genome (CP002084) are linked to IS elements and six have a cognate *rdhB* [46]. In all sequenced DEH, most *rdhA* genes and IS elements are located in the high plasticity regions (HPRs) of chromosomes. Therefore, several authors have suggested that their dissemination could be linked to intrachromosomal gene transfer events or horizontal acquisition [6,10,60,62].

## Conclusion

In this study, combination of complementary methods provided insights into the active community and functional genes present in the anoxic waters of Lake Pavin. Community analysis associated to specific enrichment methods showed the presence of 16S rRNA gene sequences that are closely related to known dehalogenating bacterial genera, mostly DEH-related *Chloroflexi*. In addition, several putative dehalogenase gene motifs were also detected in the deep anaerobic waters. Therefore, the observed data suggest that at least some of the detected phylotypes are capable of energy conservation via organohalide respiration and that these microorganisms may be widely distributed in the lake waters. Lake Pavin is thus an ideal model ecosystem to study the occurrence and the role of DEH in an uncontaminated environment. Future work will aim at isolating representative strains for these phylotypes in order to test this hypothesis and to characterize with precision the lifestyle of these fascinating microorganisms.

## Supporting Information

S1 Table Primers used for reverse transcription, pyrosequencing, qPCR and PCR approaches, and PCR annealing conditions used in this study. (PDF)

S2 Table Primers and probes used for capture experiments. (PDF)

S3 Table Statistical indices for pyrosequencing datasets determined using Panam software (http://code.google.com/p/panam-phylogenetic-annotation/) [36]. (PDF)

S4 Table Overview of the composition of genomic fragments recovered using the gene capture approach targeting DEH 16S rRNA genes and reductive dehalogenase genes. (PDF)

S5 Table Overview of the composition of genomic fragments recovered using the capture approach targeting IS911 elements. (PDF)
