# Supplementary material for: Distribution of Dehalococcoidia in the Anaerobic Deep Water of a Remote Meromictic Crater Lake and Detection of Dehalococcoidia-Derived Reductive Dehalogenase Homologous Genes
Source: PLoS One. 2016 Jan 6;11(1):e0145558. doi: 10.1371/journal.pone.0145558 (PMC4703385; doi:10.1371/journal.pone.0145558)
Supplement: S1 Table — (PDF) [file pone.0145558.s001.pdf]

S1 Table. Primers used for reverse transcription, pyrosequencing, qPCR and PCR approaches and PCR annealing conditions used in this study.

Application	Primer	Primers (5' - 3')	Annealing temperature	Targeted genes	References
**Reverse transcription**	R1492	GGYTACCTTGTTACGACTT	52°C	Bacterial 16S rRNA gene	Mofidied from [1]
**Pyrosequencing**	27F	AGRGTTTGATCMTGGCTCAG	54°C	Bacterial 16S rRNA gene	Mofidied from [1]
	Gray519R	GTNTTACNGCGGCKGCTG			[2]
**PCR**	DHC16S9F	CTAGCGGCGTGCCTTAT	56°C	*Dehalococcoidia* 16S rRNA gene	[3]
	1492R	GGYTACCTTGTTACGACTT			[1]
	Deb179F	TGTATTGTCCGAGAGGCA	50°C	*Dehalobacter* 16S rRNA gene	[4]
	Dhb1273R	CTTCCGTCTGTACCGTCCAT			[5]
	Desulfitfw	CGTTARTRGATGGATCCGC	50°C	*Desulfitobacterium* 16S rRNA gene	This study
	Dd2R	TAGCGATTCCGACTTCATGTTC			[6]
	Geo196F	GAATATGCTMCTGTATC	50°C	*Geobacter* 16S rRNA gene	[7]
	Geo825r	TACCCGCRACACCTAGTTCT			[8]
	Sug016F	GCTAAAGGATGGGGCTTTA	50°C	*Sulfurospirillum*16S rRNA gene	[9]
	Dhspm1210R	GTATCGCGTCTCTTTGTCCTA			[10]
	rdhaF2	ATTCCCTSYATGAATGACAC	50°C	Reductive dehalogenase	This study
	rdhaRev	GGCAKRCAGCAATACARTTGG			This study
	IntFw1	AAGCGGTRAARCTGGTTACAG	48°C	IS3/IS911 insertion element	This study
	IntRev1	GTAGAAGADTTCGATRTACTC			This study
	IntFw1	AAGCGGTRAARCTGGTTACAG	58°C	IS3/IS911 insertion element	This study
	IntRev2	TGAYCAGGTTTCTGGTCAA			This study
	IntFw2	CCGCGTACTTTGCCARGGAGT	50°C	IS3/IS911 insertion element	This study
	IntRev1	GTAGAAGADTTCGATRTACTC			This study

	IntFw2	CCGCGTACTTTGCCARGGAGT	53°C	IS3/IS911 insertion element	This study
	IntRev2	TGAYCAGGTTTCTGGTCAA			This study
	IntFw3	GGTRGGTATWTGCCGGATCAAG	51°C	IS3/IS911 insertion element	This study
	IntRev1	GTAGAAGADTTCGATRTACTC			This study
	IntFw3	GGTRGGTATWTGCCGGATCAAG	52°C	IS3/IS911 insertion element	This study
	IntRev2	TGAYCAGGTTTCTGGTCAA			This study
	27F	AGRGTTTGATCMTGGCTCAG	50°C	Bacterial 16S rRNA gene	Mofidied from [1]
	E1-1R	CCTACCGATTACAAGTCGGTTG		Threonine tRNA gene	This study
**qPCR**	341f	CCTACGGGAGGCAGCAG	54°C	Bacterial 16S rRNA gene	[11]
	534r	ATTACCGCGGCTGCTGGCA			[11]
	Dmonas-6	GTCGTACGGTTTCTCGCAAG	54°C	*Dehalogenimonas* 16S rRNA gene	This study
	Dmonas-D	AGTGATCCTTCTTATGAAAG			This study
	Dtes-4	GAAGAAATATGACGGTACTC	59°C	*Dehalococcoides* 16S rRNA gene	This study
	Dtes-C	CCTCAACGTCAGGAGCATC			This study

1. Lane D. 16S/23S rRNA sequencing. Nucleic acid techniques in bacterial systematics. John Wiley and Sons. New-York: Erko Stackebrandt and Michael Goodfellow; 1991. pp. 115–174.

2. Manefield M, Whiteley AS, Griffiths RI, Bailey MJ (2002) RNA stable isotope probing, a novel means of linking microbial community function to phylogeny. Applied Environmental Microbiology 68: 5367–5373. doi:10.1128/AEM.68.11.5367-5373.2002

3. Holmes VF, He J, Lee PKH, Alvarez-Cohen L (2006) Discrimination of multiple *Dehalococcoides* strains in a trichloroethene enrichment by quantification of their reductive dehalogenase genes. Applied Environmental Microbiology 72: 5877–5883. doi:10.1128/AEM.00516-06.

4. Schlötelburg C, von Wintzingerode C, Hauck R, von Wintzingerode F, Hegemann W, Göbel UB (2002) Microbial structure of an anaerobic bioreactor population that continuously dechlorinates 1, 2-dichloropropane. FEMS Microbiology Ecology 39: 229–237. doi:10.1111/j.1574-6941.2002.tb00925.x.

5. Nelson JL, Fung JM, Cadillo-Quiroz H, Cheng X, Zinder SH (2011) A role for *Dehalobacter* spp. in the reductive dehalogenation of dichlorobenzenes and monochlorobenzene. Environmental Science & Technology 45: 6806–6813. doi:10.1021/es200480k.

6. Smits TH., Devenoges C, Szynalski K, Maillard J, Holliger C (2004) Development of a real-time PCR method for quantification of the three genera *Dehalobacter*, *Dehalococcoides*, and *Desulfitobacterium* in microbial communities. Journal of Microbiological Methods 57: 369–378. doi:10.1016/j.mimet.2004.02.003.

7. Amos BK, Sung Y, Fletcher KE, Gentry TJ, Wu W-M, Criddle CS, et al. (2007) Detection and quantification of *Geobacter lovleyi* strain SZ: Implications for bioremediation at tetrachloroethene- and uranium-impacted sites. Applied Environmental Microbiology 73: 6898–6904. doi:10.1128/AEM.01218-07.

8. Anderson RT, Rooney-Varga JN, Gaw CV, Lovley DR (1998) Anaerobic benzene oxidation in the Fe (III) reduction zone of petroleum-contaminated aquifers. Environmental Science & Technology 32: 1222–1229.

9. Stolz JF, Ellis DJ, Blum JS, Ahmann D, Lovley DR, Oremland RS (1999) Note: *Sulfurospirillum barnesii* sp. nov. and *Sulfurospirillum arsenophilum* sp. nov., new members of the *Sulfurospirillum* clade of the ε-*Proteobacteria.* International Journal of Systematic Bacteriology 49: 1177–1180.

10. Daprato RC, Löffler FE, Hughes JB (2007) Comparative analysis of three tetrachloroethene to ethene halorespiring consortia suggests functional redundancy. Environmental Science & Technology 41: 2261–2269. doi:10.1021/es061544p.

11. Watanabe K, Kodama Y, Harayama S (2001) Design and evaluation of PCR primers to amplify bacterial 16S ribosomal DNA fragments used for community fingerprinting. Journal of Microbiological Methods 44: 253–262. doi:10.1016/S0167-7012(01)00220-2.
