# Supplementary material for: Distribution of Dehalococcoidia in the Anaerobic Deep Water of a Remote Meromictic Crater Lake and Detection of Dehalococcoidia-Derived Reductive Dehalogenase Homologous Genes
Source: PLoS One. 2016 Jan 6;11(1):e0145558. doi: 10.1371/journal.pone.0145558 (PMC4703385; doi:10.1371/journal.pone.0145558)
Supplement: S2 Table — (PDF) [file pone.0145558.s002.pdf]

S2 Table. Primers and probes used for capture experiments.

Aplication	Primers / probes (5' - 3')	Name	Size (pb)	Target	References
**Library construction**	CCATCTCATCCCTGCGTGTCTCCGACGACT	A adaptor key	30	-	Roche Applied Science
	CCTATCCCCTGTGTGCCTTGGCAGTCGACT	B adaptor key	30	-	
**PCR**	GGTTCTAATACGACTCACTATAGGGATCGCACCAGCGTGT	T7-A	40	Single strand capture probe	[1]
	CGTGGATGAGGAGCCGCAGTG	B	21		
	CCATCTCATCCCTGCGTGTC	454 Ti-A	20	Genomic DNA library	Roche Applied Science
	CCTATCCCCTGTGTGCCTTG	454 Ti-B	20		
**Capture 1**	GTCGTACGGTTTCTCGCAAGAGAGACAGTGGCAAACGGGT	16S_pos8	40	*Dehalococcoidia* 16S rRNA genes	This study
	TAGTTAGGTAACCTGCCTTTAAGTGGGGGATAACACTTCGAAAGA	16S_pos50	45		This study
	GCTAGTAAACGCGTGTCAGCATAGCGCGTTGAATACGTTCTCGGGCCT	16S_pos1250	48		This study
	ATATGCCTTTGAATGTCGATAAACCAATTGATTTTGGTGTAACAGAGTTTTGT	Capt_rdase	53	Reductive dehalogenase	This study
	AAAGCAGCCGCGTACTTTGCCAGGGAGTCGCTGCCCGGTACGCGATGATA	Capt_IS3_F1R1_1	50	Insertion element	This study
	GTTCCCACCGACGAAGG**Y**TGGCTGTATCTGGC**R**GG**M**CACAAGGACCTCTT	Capt_IS3_F1R1_2	50		This study
	CAGGAATTGGT**R**CATCACCGCCGCTATAGCACCAGACAGGAGGCAGTTCGA	Capt_IS3_F1R1_3	51		This study
	CGCTGCCCGGTACG**Y**GATGAT**R**AAG**R**AACTGCGGCTCGATTATCCGGT	Capt_IS3_F2R2_1	48		This study
	GTTACTGCCCCGAATAAAGT**R**TGGACAAGCGATATAACCTATGTGCCCAC	Capt_IS3_F2R2_2	50		This study

	CGTATCCGGAAGAAGCTGGGCATACGCTGCAAGCAGAAGAGGAAGTTCAA	Capt_IS3_E8E5_1	50		This study
	AATGCGCCGATGGAGAGTTTCTGGGGAACACTCAAGCAGGAGCTGGTACA	Capt_IS3_E8E5_2	50		This study
	AACGCGCCGATGGAGAGTTTCTGGGGTACACTCAAACAGGAATTGGTACA	Capt_IS3_E7_1	50		This study
	TGGCTGTAT**Y**TTGCCGGCCATAAGGATCTTTTTACTGGAGA**R**GT**Y**GTAGG	Capt_IS3_E9_1	50		This study
**Capture 2**	ATTACGCTCTGATGATGGGTGCAGCTACGATAAATTACATTGTAGGTTATCATG	RdaseH8-1	54	Reductive dehalogenase	This study
	TGCAGCCGCTACATATGGTGCTGCTCTTGTCGGTATTACCGATATTGATAGA	RdaseH8-2	52		This study
	GATGGACGACTTCATGGGCTATGGTGTACGCACGGGTGACGACATCGAAAAGTTTT	C5-1	56	Reductive dehalogenase	This study
	CTGGGCGTGCTGGGGCTGGCTCTGCTCCTCTTCTCTCTCCAGAACTACGTAGGTTC	C5-2	56		This study
	GAGGATGAAGCTCTGGAAAGCCGCTGCCTCACCGCTACCATGCTCACCCTC	RdaseH8-3	51	Hypothetical protein	This study
	ATCTTCGGTCTAAGCTGCTTACCTTCCGCCTGCACAACCTGCTCAAACTTAA	RdaseH8-4	52		This study
	GATGGCCAGGTCAAACGGCAGAGTAAAGTACAGGTCGGCGAAAACCTTGTC	H9-1	51	Hypothetical protein	This study
	GATTCTCTATCTCCACCCAGATGCAGTCCTTAACGTCAAGCATCAGCACGC	H9-2	51		This study

The 40 to 56-mers probes designed are specific or degenerated (letters in bold, IUPAC standard ambiguity codes for nucleotides).
Capture 1 probes were used for initial enrichment of DEH genomic DNA.
Capture 2 probes were used for retrieving specific genome fragments.

1. Gnirke A, Melnikov A, Maguire J, Rogov P, LeProust EM, Brockman W, et al. (2009) Solution hybrid selection with ultra-long oligonucleotides for massively parallel targeted sequencing. Nature Biotechnology 27: 182–189. doi:10.1038/nbt.1523.
