# Supplementary material for: Distribution of Dehalococcoidia in the Anaerobic Deep Water of a Remote Meromictic Crater Lake and Detection of Dehalococcoidia-Derived Reductive Dehalogenase Homologous Genes
Source: PLoS One. 2016 Jan 6;11(1):e0145558. doi: 10.1371/journal.pone.0145558 (PMC4703385; doi:10.1371/journal.pone.0145558)
Supplement: S3 Table — (PDF) [file pone.0145558.s003.pdf]

S3 Table. Statistical indexes for pyrosequencing datasets determined using Panam software (http://code.google.com/p/panam-phylogenetic-annotation/) [1].

Sample	Depth (m)	No. of reads	OTUs[a]	Chao1	Shannon	ACE	Coverage (%)
cDNA	58	3442	209	365	2.78	380	97.04
	60	2269	170	273	2.64	304	96.34
	63	3208	259	500	3.08	518	95.82
	68	2174	246	453	3.70	425	94.43
	80	2131	399	687	4.63	753	90.29
gDNA	52	2654	280	465	4.05	529	95.06
	58	2873	263	492	3.94	493	95.58
	60	2596	305	505	4.16	595	94.07
	63	2747	390	732	4.45	764	92.61
	68	2261	315	542	4.09	570	92.92
	80	1399	409	704	5.20	724	84.92

[a]Clustered at 97% identity; ACE, abundance-based coverage estimator; No., number.

1. Taib N, Mangot J-F, Domaizon I, Bronner G, Debroas D (2013) Phylogenetic affiliation of SSU rRNA genes generated by massively parallel sequencing: New insights into the freshwater protist diversity. PLoS ONE 8: e58950. doi:10.1371/journal.pone.0058950
