# Supplementary material for: Distribution of Dehalococcoidia in the Anaerobic Deep Water of a Remote Meromictic Crater Lake and Detection of Dehalococcoidia-Derived Reductive Dehalogenase Homologous Genes
Source: PLoS One. 2016 Jan 6;11(1):e0145558. doi: 10.1371/journal.pone.0145558 (PMC4703385; doi:10.1371/journal.pone.0145558)
Supplement: S4 Table — (PDF) [file pone.0145558.s004.pdf]

S4 Table. Overview of the composition of genomic fragments recovered using the gene capture approach targeting DEH 16S rRNA genes and reductive dehalogenase genes.

Target	Contig name	Size (bp)	Contig composition	Gene sequence	Locations	Closest relative	Accession number*	Query cover	Identity**
16S rRNA genes	Contig_DmonasA2	2,140	16S rRNA gene	full	272-1,760	*Dehalogenimonas alkenigignens* SBP1	JQ994267	96%	98%
			tRNA Ile	full	1,969-2,055	*Dehalogenimonas lykanthroporepellens* BL-DC-9	CP002084	100%	94%
	Contig_DtesH9	2,573	16S rRNA gene	partial	1-1,387	uncultured bacterium clone FGL12_B47	FJ437892	97%	98%
			Hypothetical protein	full	1,612-2,340	*Dehalococcoides mccartyi* VS DhcVS_900	ACZ62020	99%	52%
			Acylphosphatase	partial	comp(2346-2573)	*Dictyoglomus turgidum* DSM 6724	YP_002353284	98%	42%
	Contig_H9H2	990	16S rRNA gene	partial	1-363	bacterium clone FGL12_B47	FJ437892	100%	98%
			Hypothetical protein	partial	629-990	*D. mccartyi* VS DhcVS_900	ACZ62020	99%	58%
	Contig_H9H4	1,512	16S rRNA gene	partial	1-347	bacterium clone FGL12_B47	FJ437892	100%	98%
			Hypothetical protein	full	582-1,309	*D. mccartyi* VS DhcVS_900	ACZ62020	99%	52%
			Acylphosphatase	partial	comp(1,315-1,512)	*D. turgidum* DSM 6724	YP_002353284	94%	44%
	Contig_H9E2	1,298	hypothetical protein	full	582-1,309	*D. mccartyi* VS DhcVS_900	ACZ62020	99%	52%
			Acylphosphatase	full	comp(964-1,245)	*D. turgidum* DSM 6724	YP_002353284	92%	44%
	Contig_H9E9	1,278	Hypothetical protein	partial	1-679	*D. mccartyi* VS DhcVS_900	ACZ62020	99%	53%
			Acylphosphatase	full	comp(686-967)	*D. turgidum* DSM 6724	YP_002353284	92%	48%
	Contig_H9E1	1,088	Hypothetical protein	partial	1-250	*D. mccartyi* VS DhcVS_900	ACZ62020	97%	47%
			Acylphosphatase	full	comp(268-540)	*D. turgidum* DSM 6724	YP_002353284	92%	44%
			tRNA Thr	full	890-964	*D. mccartyi* VS	CP001827	98%	95%
			tRNA Tyr	full	987-1,072			100%	88%
Reductive dehalogenase	Contig_RdaseH8	2,538	Tetrachloroethene reductive dehalogenase	partial	1-1,056	*Heliobacterium modesticaldum* Ice1	YP_001680129	95%	55%
			Hypothetical protein	partial	comp(1,636-2,538)	*D. mccartyi* VS DhcVS_220	ACZ61382	99%	62%
	Contig_RdaseD8E3	3,258	Hypothetical protein	partial	comp(1-434)	*D. mccartyi* VS DhcVS_300	ACZ61461	98%	60%
			Hypothetical protein	full	comp(438-785)	*D. mccartyi* VS DhcVS_301	ACZ61462	94%	48%
			Hypothetical protein	full	1,024-1,797	*Anaerolinea thermophila* UNI-1 ANT_31770	YP_004175801	90%	32%
			Hypothetical protein	partial	comp(2,707-3,258)	*D. mccartyi* VS DhcVS_220	ACZ61382	97%	51%
	Contig_RdaseD8E12	2,228	Hypothetical protein	partial	comp(868-2,228)	*D. mccartyi* VS DhcVS_220	ACZ61382	89%	57%

* accession number of the protein for the genes encoding proteins
** % identity at the protein level for genes encoding proteins
comp, complement
