# Supplementary material for: Distribution of Dehalococcoidia in the Anaerobic Deep Water of a Remote Meromictic Crater Lake and Detection of Dehalococcoidia-Derived Reductive Dehalogenase Homologous Genes
Source: PLoS One. 2016 Jan 6;11(1):e0145558. doi: 10.1371/journal.pone.0145558 (PMC4703385; doi:10.1371/journal.pone.0145558)
Supplement: S5 Table — (PDF) [file pone.0145558.s005.pdf]

S5 Table. Overview of the composition of genomic fragments recovered using the capture approach targeting IS911 elements.

Cluster	Contig name	Contig size (bp)	Contig composition		Gene sequence	Location	Closest relative	Accession number*	Query cover	Identity**
Cluster 1	Contig_RdaseC5	2,524	Transposase IS3/IS911	ORF-A	full	111-428	*Dehalogenimonas lykanthroporepellens* BL-DC-9	YP_003757922	99%	70%
				ORF-B	full	425-1,285		YP_003757923	99%	75%
			Anchoring protein (RdhB)		full	comp(2,028-2,315)	*Dehalococcoides mccartyi* BTF08	YP_007485820	100%	52%
			Reductive dehalogenase		partial	comp(2,330-2,524)	uncultured bacterium	BAI47792	96%	59%
	Contig_RdaseC5F9	2,911	Anchoring protein (RdhB)		full	comp(48-329)	*D. lykanthroporepellens* BL-DC-9	YP_003759108	96%	52%
			Reductive dehalogenase		full	comp(376-1,711)	uncultured bacterium	BAI47792	95%	55%
			Hypothetical protein DUF2196		full	comp(2,220-2,414)	*Sporosarcina newyorkensis*	WP_009498944	95%	79%
	Contig_F1R1F4	2,608	Hypothetical protein		partial	comp(1-565)	*D. lykanthroporepellens* BL-DC-9	YP_003758885	96%	29%
			Transposase IS3/IS911	ORF-A	full	1,559-1,876		YP_003757922	99%	70%
				ORF-B	partial	1,873-2,608		YP_003758963	99%	77%
Cluster 2	Contig_F1R1A02	2,941	Cell division protein FtsK		partial	171-1,641	*D. lykanthroporepellens* BL-DC-9	YP_003757801	98%	63%
			Hypothetical protein		partial	comp(1,655-2,261)	*Haliscomenobacter hydrossis* DSM 1100	YP_004450849	100%	53%
			Transposase IS3/IS911	ORF-A	full	2,393-2,711	*D. lykanthroporepellens* BL-DC-9	YP_003757922	99%	66%
				ORF-B	partial	2,708-2,941		YP_003758871	100%	67%
	Contig_F1R1G11	2,460	Cell division protein FtsK		partial	1-449	*D. lykanthroporepellens* BL-DC-9	YP_003757801	86%	58%
			Hypothetical protein		partial	comp(463-1,069)	*H. hydrossis* DSM 1100	YP_004450849	100%	53%
			Transposase IS3/IS911	ORF-A	full	1,202-1,519	*D. lykanthroporepellens* BL-DC-9	YP_003757922	99%	66%
				ORF-B		1,516-2,376		YP_003758963	99%	70%
	Contig_F1R1B4	2,037	Ferredoxin		partial	comp(1-1,387)	uncultured *Desulfobacterium* sp.	CBX26965	99%	49%
			Transposase IS3/IS911	ORF-A	full	1,507-1,824	*D. lykanthroporepellens* BL-DC-9	YP_003757922	99%	68%
				ORF-B	partial	1,821-2,037		YP_003758871	99%	65%
	Contig_F1R1A2T7	2,128	Transposase IS3/IS911	ORF-A	full	888-1,205	*D. lykanthroporepellens* BL-DC-9	YP_003757922	99%	73%
				ORF-B	partial	1,202-2,062		YP_003758963	99%	71%

	Contig_F1R1pl1A10	3,228	PAS/PAC sensor signal transduction histidine kinase		partial	1-256	*D. mccartyi* GY50	YP_008855801	92%	63%
			HicB family protein		full	733-1,098	*Alicyclobacillus pohliae*	WP_018131235	96%	48%
			Hypothetical protein PTH_2558		full	comp(1,189-1,405)	*Pelotomaculum thermopropionicum* SI	YP_001213108	95%	60%
			Transposase IS3/IS911	ORF-A	full	2,554-2,871	*D. lykanthroporepellens* BL-DC-9	YP_003757922	99%	67%
				ORF-B	partial	2,867-3,228		YP_003758871	98%	70%
Cluster 3	Contig_F1R1A12	2,654	Transposase IS3/IS911	ORF-A	partial	1-76	*D. lykanthroporepellens* BL-DC-9	YP_003757922	94%	88%
				ORF-B	full	76-936		YP_003758963	99%	77%
			Beta-lactamase		partial	1,628-2,654	*Desulfatibacillum alkenivorans*	YP_002433949	98%	43%
	Contig_F1R1_3F5	3,293	Ferredoxin		full	comp(294-931)	*Desulfotomaculum kuznetsovii*	YP_004518429	98%	50%
			Putative transposase		partial	comp(1,221-1,873)	*D. mccartyi* GY50	YP_008856417	98%	45%
			Transposase IS3/IS911	ORF-A	full	1,920-2,235	*D. lykanthroporepellens BL-DC-9*	YP_003757922	98%	69%
				ORF-B	full	2,231-3,095		YP_003758963	99%	77%
	Contig_F3R1D8	2,119	Transposase IS3/IS911	ORF-B	partial	1-494	*D. lykanthroporepellens BL-DC-9*	YP_003757923	98%	80%
			ADP-ribosylation/Crystallin J1		full	comp(526-1,607)	*Coleofasciculus chthonoplastes*	WP_006101108	98%	53%
			Hypothetical protein		full	comp(1,615-2,052)	*Oscillatoria nigro-viridis*	YP_007113822	97%	56%
	Contig_F2R2C11	1,963	Transposase IS3/IS911	ORF-B	partial	1-748	*D. lykanthroporepellens* BL-DC-9	YP_003758085	99%	79%
			Hypothetical protein		partial	comp(1,616-1,963)	*Archaea*	WP_018033988	98%	49%
	Contig_F1R1A6	1,360	Transposase IS3/IS911	ORF-B	partial	1-498	*D. lykanthroporepellens* BL-DC-9	YP_003758963	99%	79%
	Contig_F1R1E9	2,746	Glycosyl transferase group 1		partial	1-438	*Caldicellulosiruptor kristjanssonii*	YP_004027490	78%	33%
			Transposase IS3/IS911	ORF-A	full	646-961	*D. lykanthroporepellens* BL-DC-9	YP_003757922	96%	73%
				ORF-B	full	958-1,821		YP_003758871	99%	75%
			Hypothetical protein		partial	1,885-2,746	*Pyrococcus* sp. ST04|	YP_006354137	98%	35%
	Contig_F1R1-3F3	3,511	Transposase IS3/IS911	ORF-A	full	254-569	*D. lykanthroporepellens* BL-DC-9	YP_003757922	96%	73%
				ORF-B	full	566-1,429		YP_003758963	99%	74%
			Hypothetical protein		full	1,498-2,681	*Pyrococcus* sp. ST04|	YP_006354137	99%	38%
	Contig_F1R1A10	1,454	Transposase IS3/IS911	ORF-B	partial	1-375	*D. lykanthroporepellens* BL-DC-9	YP_003757923	99%	78%
			Site specific recombinase		partial	comp(563-1,454)	*D. mccartyi* GY50	YP_008855351	70%	31%

Cluster 4	Contig_F1R1pl2C4	3,542	Phosphoribosylaminoimidazole-succinocarboxamide synthase		partial	comp(1-241)	*D. lykanthroporepellens* BL-DC-9	YP_003758285	89%	72%
			tRNA Gln		full	380-453	*Tistrella mobilis*	CP003236	100%	89%
			tRNA Glu		full	474-554	*D. mccartyi GY50*	CP006730	100%	91%
			Phage integrase site specific recombinase		partial	comp(660-960)	*D. mccartyi* 195	YP_182174	97%	63%
			Transposase IS3/IS911	ORF-A	full	1,008-1,322	*D. lykanthroporepellens* BL-DC-9	YP_003757922	99%	71%
				ORF-B	full	1,322-2,182		YP_003758871	99%	76%
Cluster 5	Contig_F1R1pl1D6	2,086	Transposase IS3/IS911	ORF-A	full	comp(981-1,290)	*D. lykanthroporepellens* BL-DC-9	YP_003757922	97%	68%
				ORF-B	full	comp(125-981)		YP_003758963	99%	79%
			Hypothetical protein DUF45		full	1,361-2,075	*Dehalococcoides* sp. VS	YP_003329660	88%	35%

* accession number of the protein for the genes encoding proteins
** % identity at the protein level for genes encoding proteins
comp, complement
